# Cognitive-behavioral therapy on psychological stress and quality of life in subjects with pulmonary tuberculosis: a community-based cluster randomized controlled trial

**DOI:** 10.1186/s12889-022-14631-6

**Published:** 2022-11-24

**Authors:** Xiaowei Zuo, Zongmei Dong, Peng Zhang, Pan Zhang, Xianghua Zhu, Cheng Qiao, Yongjie Yang, Peian Lou

**Affiliations:** 1 Department of psychiatry, Xuzhou Oriental People’s Hospital, 379 Tongshan Road, Xuzhou, 221004 Jiangsu Province China; 2 Department of Control and Prevention of Chronic Non-communicable Diseases, Xuzhou Center for Disease Control and Prevention, 142 West Erhuan Road, Xuzhou, 221006 Jiangsu Province China

**Keywords:** Cognitive-behavioral therapy, Pulmonary tuberculosis, Anxiety, Depression, Quality of life

## Abstract

**Background:**

Anxiety and depression are two common psychological disorders in patients with pulmonary tuberculosis. We aimed to explore the effects of cognitive-behavioral therapy (CBT) on psychological stress and quality of life in patients with pulmonary tuberculosis.

**Methods:**

From September 2018 to November 2018, 20 communities (461 participants in total) were randomly assigned in an intervention or control group following a two-level cluster random design. The intervention group underwent CBT for 2 months, whereas the control group received routine follow-up. Anxiety, depression, and quality of life were assessed using the Patient Health Questionnaire-9 (PHQ-9), General Anxiety Disorder questionnaire (GAD-7), and 36-Item Short-Form Health Survey (SF-36) scales, respectively. Comparisons between the two groups were conducted using independent samples t-tests, and differences between the two groups before and after treatment were analyzed using paired samples t-tests.

**Results:**

There were a total of 454 participants in the final analysis. After 2 months of CBT intervention, the CBT group had a GAD-7 score that was 1.72 lower than the control group (1.47–1.99, *p* < 0.001), a PHQ-9 score of the CBT group that was 2.05 lower than that of the control group (1.74–2.37, *p* < 0.001). The CBT group had a total SF-36 score that was 10.7 lower than that of the control group (95% CI: 7.9–13.5, p < 0.001). In patients with different degrees of anxiety and depression, only those in the intervention group who had mild and moderate anxiety and depression symptoms showed a significant reduction in anxiety and depression scores following the intervention.

**Conclusions:**

CBT can relieve anxiety, and depression symptoms and increase the quality of life in subjects with pulmonary tuberculosis.

**Trials registration:**

ChiCTR-TRC-12001958 Date of Registration: 22/02/2012.

## Background

Tuberculosis is caused by *Mycobacterium tuberculosis* (Mtb) and is a widespread and fatal chronic infectious disease. It is spread by droplets from the throat and lungs of acutely infected people. At present, approximately one-third of the global population has previously been infected with Mtb. Mtb carriers may remain infectious for life, although there is only a 10 to 20% probability that an individual transition from the incubation period to the active period [1]. Nevertheless, the incidence of tuberculosis is ranked at first position among all infectious diseases in the world and is one of the top ten causes of death [2]. In 2018, there were 866,000 pulmonary tuberculosis patients in China, which accounted for 1/11 of global pulmonary tuberculosis patients; 15,700 of these patients died of the disease. Tuberculosis remains a major public health problem globally [2]. 14,725 active pulmonary tuberculosis patients were registered in Xuzhou from 2016 to 2020. Because patients with pulmonary tuberculosis require a minimum of 6 months of regular treatment, psychological problems occur frequently [2, 3]. Anxiety and depression are two common psychological symptoms experienced by patients with pulmonary tuberculosis [4, 5]. Pulmonary tuberculosis patients who experience anxiety or depression have decreased treatment efficacy, physical skills, and reduced quality of life [6–8].

Psychological interventions can improve treatment compliance, quality of life, and treatment efficacy by reducing the psychological pressure on patients with tuberculosis [9, 10]. However, current existing treatment strategies for tuberculosis patients are more focused on chemotherapy treatments of *Mycobacterium tuberculosis* and disregard psychological treatments [6]. Cognitive-behavioral therapy (CBT) has been recommended as the preferred non-drug therapy for anxiety and depression in adults [11] and has been implemented successfully for the psychological treatment of chronic diseases, such as chronic obstructive pulmonary disease [12], asthma [13], and diabetes [14].

CBT was a short-term psychotherapy method, which mainly removed bad cognition by changing the patient’s thinking, belief and behavior, so as to eliminate negative emotions and behaviors. The use and impact of CBT has been steadily expanding since the advent of this method in the 1950s. The central premise of this therapeutic approach, pioneered by Baker [15] and Ellis [16], was that people’s cognitions had a controlling influence on emotions and behaviors, and that the behaviors strongly influenced thought patterns and emotions. CBT was a problem-oriented therapy, usually in a short-term form. Treatment for less complex depression and anxiety disorders usually lasted 5–20 times. The duration of each treatment was 45–50 minutes. However, if there were concurrent symptoms or if the patient had symptoms of long-term treatment or refusal to treatment, more than 20 treatments may be required. At present, CBT has been widely used in the treatment of depression or anxiety disorder in clinical practice, and were effective.

However, there have not yet been any reports on the use of CBT to treat psychological disorders in pulmonary tuberculosis patients. The directly observed treatment and short-course chemotherapy (DOTS) strategy recommended by the World Health Organization [17] required community-based general practitioners to supervise the daily medication administration of tuberculosis patients. If general practitioners can learn and deliver CBT combined with DOTS, it will help to reduce the incidence of psychological disorders and improve the quality of life of pulmonary tuberculosis patients. In our randomized controlled trial, CBT was delivered by general practitioners to patients with pulmonary tuberculosis for 8 weeks to explore the effectiveness of CBT for treating psychological disorders and improving the quality of life in patients with pulmonary tuberculosis.

## Methods

### Study design

We used a single-blind randomized controlled trial design. To facilitate intervention, we sampled from communities, rather than individuals with TB. Communities were selected using a random sequence, based on a prevalence of depression and anxiety in tuberculosis patients of 46.3 and 47.2%, respectively [11]. To ensure sufficient sample size, we calculated that with a prevalence of depression in patients with tuberculosis of 45%, each community would have at least 45 patients. Taking into account exclusions based on eligibility, we estimated that each community would have at least 50 patients. The study ran from September 2018 to November 2019. In August 2018, 183 pulmonary tuberculosis patients who required treatment for at least 4 months were recruited for the baseline assessment, and we found that 85 patients (44 in the intervention group and 41 in the control group) had anxiety or depression. From September 2018 to August 2019, newly diagnosed pulmonary tuberculosis patients who were receiving treatment were assessed within 3 days of receiving a report from community general practitioners. After patients received 2 months of CBT intervention, post-intervention assessments were performed within 5 days. All assessments took place at community health service stations and participants received a gift after each research. The Consolidated Standards of Reporting Trials (CONSORT) guidelines were followed by this study [18].

### Participants

Patients were included if they had been diagnosed with pulmonary tuberculosis and were registered under management according to the guidelines on diagnosis and treatment of pulmonary tuberculosis [19]; had a Patient Health Questionnaire-9 (PHQ-9) score of ≥5 or a General Anxiety Disorder questionnaire (GAD-7) score of ≥5; and had a normal cognitive function at entry. Exclusion criteria were a GAD-7 score or PHQ-9 score of < 5; psychiatric history or cognitive impairment; had taken or were taking antipsychotic drugs; history of panic disorder or bipolar depression; participation in any other psychological intervention trial; or medically unstable (e.g., cancer, stroke, cardiovascular disease, chronic obstructive pulmonary disease, and severe psychosis). We excluded participants who had a life expectancy of less than 1 year, were unwilling to participate in the study, were unable to attend regular sessions (being absent for more than two sessions), or had experienced severe crises or stress before the study.

Participants were considered lost to follow-up if they could not be contacted or if they moved to another region, withdrew from the study, refused to proceed, had invalid data, or were unable to complete the study.

### Ethical consideration

The present study was conducted under protocols approved by the Xuzhou Center for Disease Control and Prevention and the Xuzhou of Medical Sciences Ethics Committee (Approval No. 2012010). Written informed consent was obtained from all participants. The trial was registered with the Chinese Clinical Trials Registry (reference: ChiCTR-TRC-12001958) and conducted according to the 2000 revised version of the Helsinki Declaration.

### Sample size

Using a two-level cluster random design [20], a total of 2 N communities were selected and randomly assigned to an intervention or control group. The probability of Type I error was 0.05 and the test power was 0.8. A total of 20 subjects were included in each community. On the basis of previous reports [12, 14], we assumed that (μ1 − μ2) = 2.0 would be a meaningful difference in depression values between the two groups following the intervention. The total variance (σ) was 5, and the intra-group correlation coefficient ρ was estimated to be 0.05, according to the formula:

$$n=\frac{2{\left({Z}_{1-\alpha /2+{Z}_{1-\beta }}\right)}^2{\sigma}^2\left[1+\left(n-1\right)\rho \right]}{{\left({\mu}_1-{\mu}_0\right)}^2}$$

We concluded that each group needed at least 191 participants from 10 communities. Accounting for an estimated 10% rejection rate, we calculated that each group would require at least 210 patients.

### Randomization and masking

The community with more than 50 registered pulmonary tuberculosis patients was used to generate a random number sequence on a computer, and 20 communities were selected. Communities were randomly allocated by a research statistician who had no contact with the participants. We randomly assigned (1:1) the 20 communities to receive either CBT plus usual care (intervention group) or usual care only (control group) using a random digit table and informed the general practitioners of this assignment at baseline. The general practitioners scheduled all study intervention appointments for the participants. Investigators and statisticians were blinded for grouping. The participants were recruited by our team members and general practitioners. The general practitioners were responsible for notifying and convening the recruited members, our team members were responsible for explaining the patient instructions to the participants and signing the informed consent.

### Interventions

#### Usual care (all participants)

The control group only received the usual care from their primary healthcare service. Usual care followed DOTS, which was carried out in a unified approach throughout the country. The standard requirements of DOTS were: to provide patients with the most effective treatment; for general practitioners to deliver medicine to the patient’s hand to observe the patient taking the medicine before leaving; to confirm that the patient will attend regular follow-up visits, take medicine regularly, and undergo regular sputum examinations; and to ensure patients take medicine according to regulations until recovery. General practitioners also provided each patient with advice on ways to improve quality of life, diet, exercise, prevention of complications, drug side effects, and medication during follow-up appointments [17].

### CBT

#### General practitioner training

Twenty general practitioners (18 from community health service stations and two from community health service centers) participated in a 3-day CBT skills training session that totaled 8 hours. The training content was formulated concerning relevant documents [21] and included supportive psychotherapy, cognitive intervention, relaxation training, and behavior correction. Lesson 1 introduced the importance of the definition, etiology, infectivity, and treatment of pulmonary tuberculosis to eliminate the psychological pressure on patients and build confidence in overcoming the disease. Lesson 2 introduced the concept and treatment process of CBT. Lesson 3 considered the symptoms and concepts of anxiety and depression, the relationships between anxiety, depression, and tuberculosis, and how these affect treatment compliance and the quality of life of patients. In lesson 4, general practitioners learned how to conduct interviews and listen to pulmonary tuberculosis patients with enthusiasm and patience. This involved understanding and patiently answering questions raised by patients to convince them that all questions related to pulmonary tuberculosis can be answered effectively. Lesson 5 included evaluating the degree of each patient’s anxiety and depression, determining the causes, identifying the methods to solve problems, selecting a method to practice according to the patient’s situation, and evaluating the effect. Lesson 6 involved learning how to improve the poor habits and behaviors of patients and understanding the importance of a balanced diet, exercise, quitting smoking, and restricting alcohol. Learning 5A (Ask, Advise, Assess, Assist, and Arrange) intervention to quit smoking [22]. Lesson 7 introduced pranayama, progressive muscle relaxation, and meditation. Lesson 8 reviewed the course content and ways to teach patients how to record practice situations. It also included psychological preparation for persistent symptoms and how to deal with relapses. The Cognitive First Aid Rating Scale (CFARS) was used to evaluate the academic performance of the general practitioners [23], which ranged from 0 (lowest skill and ability) to 6 points (highest skill and ability), and ≥ 4 points reflected a professional level of skill and ability.

### Implementation of the CBT program for participants

We formulated a detailed topic outline for each class and the general practitioners were required to explain the outline for at least 40 minutes. At the beginning of the second class, patients were asked about the completion of the assignment from the first class. After each class, general practitioners discussed with the patients for 10–15 minutes. General practitioners assigned homework to patients and instructed them on how to record and complete the homework. Each lesson was recorded on a mobile phone and uploaded. Lesson one: introduce the therapists and treatment process to patients, so that patients can understand the definition, etiology, treatment, and rehabilitation of; The concept and symptoms of anxiety and depression and their relationship with pulmonary tuberculosis. Homework: analyze the causes of pulmonary tuberculosis, the impact on your life, and your understanding of the prognosis. Lesson two: understand the concept, treatment process, and purpose of cognitive behavioral therapy; The relationship between thinking, feeling, and behavior, the wrong way of thinking and cognition, and the causes. Homework: What are the current perceptions of pulmonary tuberculosis, which are wrong and which are correct? Lesson three: evaluate the anxiety and depression of each patient, help the patient analyze the causes of these symptoms and find solutions to the problems. Homework: analyze the causes of anxiety and depression. Lesson four: Identify negative cognition and thinking. Help patients understand and recognize irrational cognition and irrational automatic thinking that cause anxiety and depression, such as “It’s really unlucky, why do I have this disease” and “I will have no good life after I have this disease”. Homework: What negative cognition and thinking do you have? Lesson five: Promote emotional expression, formulate daily imagination training according to the treatment of patients, and improve the sense of expectation for the future by looking forward to a better tomorrow, 2–3 times a day, 15–20 minutes a time. At the same time, promote the expression of negative emotions of patients, and vent their negative emotions by confiding, shouting in places where nobody is, listening to music, playing games, etc. Homework: Find one or more ways to express negative emotions. Lesson six: reconstruct the cognitive model and reconstruct the cognitive model based on reality and conducive to solving problems. “Some things are not as bad as I imagined, but just some troubles”, and “I can’t influence others’ views on me. The most important thing is that I like myself”. Homework: Do you actively face various problems and pressures in life without negative emotions? Promote emotional expression, formulate daily imagination training according to the treatment of patients, and improve the sense of expectation for the future by looking forward to a better tomorrow, 2–3 times a day, 15–20 minutes at a time. At the same time, promote the expression of negative emotions of patients, and vent their negative emotions by confiding, shouting in places where nobody is, listening to music, playing games, etc. Homework: Find one or more ways to express negative emotions. Lesson seven: Introduce the patient to pranayama, progressive muscle relaxation, and meditation. The doctor first demonstrates, and then the patient practices under the guidance of the doctor. Homework: learn these three relaxation methods, practice at least twice a day, about 30 minutes each time, and make records. Lesson eight, the course was reviewed, and answers to patients’ questions from each class were collected and generated. Questions that could not be answered were reported to our study group. Our study group answered the patients’ questions over the phone. General practitioners advised patients to be psychologically prepared for persistent symptoms and discussed ways to cope with recurrences. All courses were completed within 1 month and strengthened again in the second month. After completing the course, the patients received a small gift of appreciation. Course completion by general practitioners was included in the assessment of basic public health services.

### Outcomes

The endpoint was the 2-month follow-up result of each participant. The primary outcomes were anxiety and depression symptoms at baseline and 2 months, which were assessed using the PHQ-9 and GAD-7, respectively. The GAD-7 [24] was used to screen for generalized anxiety and to evaluate the severity of symptoms. It comprised seven items, with a total score ranging from 0 to 21 points, where a higher score indicates greater symptom severity. A score of 0 to 4 points indicates no anxiety, 5 to 9 points indicates mild anxiety, 10 to 14 points indicates moderate anxiety and 15 to 21 points indicates severe anxiety. Cronbach’s alpha for the GAD-7 was 0.84 [25].

The PHQ-9 [26] was used to screen for depression and assess symptom severity. The form comprised nine items, with a total score ranging from 0 to 27 points. A higher score was indicative of greater symptom severity. A score of 0 to 4 points indicated no depression, 5 to 9 points indicated mild depression, 10 to 14 points indicated moderate depression, 15 to 19 points indicated heavy depression, and 20 to 27 points indicated severe depression. The PHQ-9 has been used previously for the evaluation of depression in pulmonary tuberculosis patients. Cronbach’s alphas for the PHQ-9 was 0.82 [27].

The secondary outcome was quality of life at baseline and 2 months, which was measured using the Medical Outcomes Study 36-Item Short-Form Health Survey (SF-36) [28]. The scale included eight dimensions: physiological function, role-physical, bodily pain, general health, vitality, social function, role-emotional, and mental health. A higher score reflected the better quality of life. The scale has been shown to have high reliability and validity for measuring the quality of life of pulmonary tuberculosis patients [28]. Cronbach’s alphas for the SF-36 was 0.87 [29].

### Other demographic and clinical variables

A self-designed questionnaire was used to collect the following information: sex, age, year of education, ethnicity, occupation, medication, smoking, drinking, and body mass index (BMI; calculated by dividing weight [kg] by height [m^2^]).

### Quality and reliability of the intervention

The training was unified across all participants in the study. The interventions and questionnaires were formulated by referring to previous literature and consulting psychological experts and were optimized using a pilot survey. The quality of the intervention was evaluated via videos uploaded by general practitioners.

Each general practitioner managed an average of 11.5 patients (median 9, range 6–17). Two clinical psychologists evaluated the completeness and reliability of CBT implementation by general practitioners using the Mentor Approach for Promoting Patient Self-Care [30] on the basis of recordings. Following the evaluations, the general practitioners were provided with feedback to revise the content of the lectures. The range of scores was 1–8 points, where 4–5 points indicated medium, and 6, 7, and 8 points indicated good, very good, and excellent.

### Statistical analysis

The completeness, rationality, logic, and consistency of the survey data for the assignment of each variable in the survey form were audited. Double entry and consistency checks ensured the accuracy of the data.

All analyses were performed using SPSS Statistics Version 16.0 (IBM Corporation, US). Continuous variables were presented as means ± standard deviations and were analyzed using parametric or nonparametric tests depending on whether the data were normally distributed. Categorical variables were presented as percentages and compared using chi-square tests. Paired t-tests were used to compare between-group differences of continuous variables before and after the intervention. Anxiety and depression were analyzed using subgroup analysis. A *p* < 0.05 indicated statistical significance. The size of the between-groups effect was represented by Hedges g, and the size of the within-group effect was represented by Cohen’s d. An effect of 0.2–0.5 was considered small, 0.5–0.8 was medium, and 0.8 or more was large [31].

## Results

### General characteristics of participants and flow

**Fig. 1:**
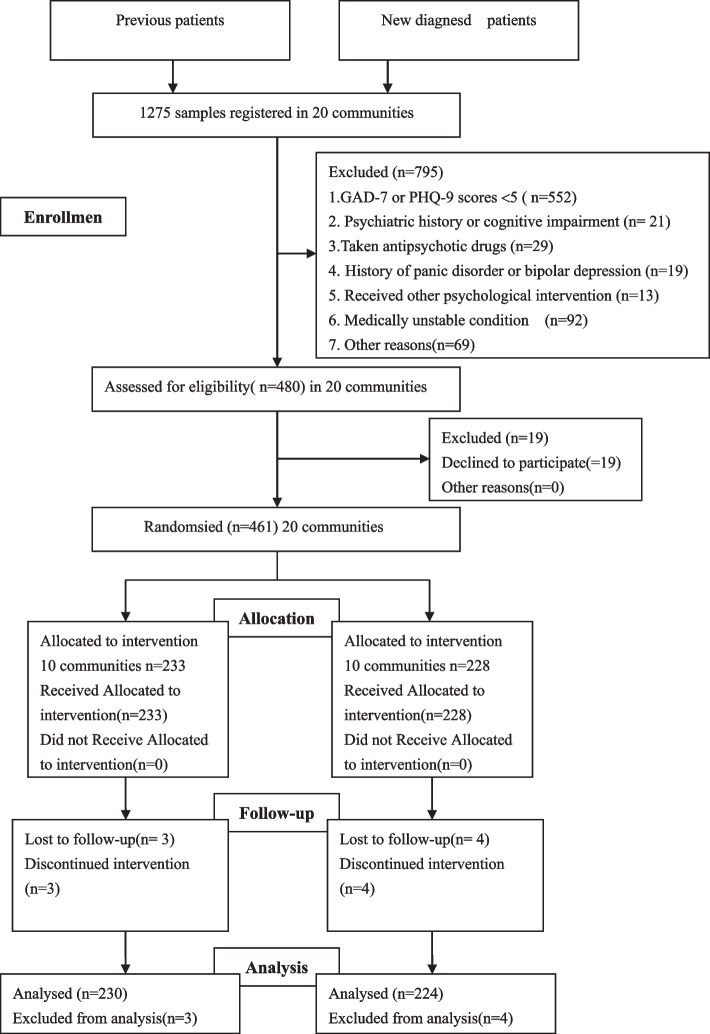
Consolidated Standards of Reporting Trials flow diagram of the participants

Figure 1 shows the CONSORT diagram of the flow of participants. A total of 1275 pulmonary tuberculosis patients participated in the baseline survey. Among them were 461 patients with anxiety or depression scores ≥5, of which 233 cases were in the intervention group and 228 cases were in the control group. In the intervention group, two cases refused CBT, one case was not followed up within 5 days of completing 2 months of CBT, and thus the ninth day follow-up was excluded from the analysis. In the control group, one case was transferred to another town following treatment for 1.5 months, and three cases were not followed up within 5 days of completing 2 months of usual care. A total of 454 patients were included in the final analysis, which comprised 230 patients from 10 communities who were assigned to the intervention group and 224 patients from 10 communities who were assigned to the control group. All tuberculosis patients were Han farmers. There were no statistically significant differences between the two groups in terms of gender, age, years of education, drug treatment, smoking, or drinking (Table 1).

**Table 1:** Comparisons between the intervention and control groups (x ± s, n [%])

Items	Intervention group	Control group	χ^2^or t	P
Gender (male)	153	151	0.041	0.84
Age	41.31 ± 10.27	41.94 ± 9.69	0.672	0.502
Years of education	8.96 ± 3.23	8.58 ± 3.64	1.177	0.240
BMI	21.76 ± 2.13	21.71 ± 2.20	0.246	0.806
Smoking	68(29.56%)	65(29.02%)	0.016	0.898
Drinking	83(36.09%)	81(36.16%)	< 0.001	0.987
Family history	10(4.35%)	7(3.13%)	0.471	0.493
Drug Treatment	230(100%)	224(100%)	–	–
GAD-7	7.57 ± 5.27	7.62 ± 5.41	0.219	0.826
PHQ-9	8.13 ± 6.01	7.98 ± 5.84	0.270	0.788
SF-36	58.46 ± 12.71	59.11 ± 13.25	0.533	0.594

Values are presented as means ± standard deviations and number (%)

BMI: body mass index; GAD-7: Generalized Anxiety Disorder-7; PHQ-9: Patient Health Questionnaire-9; SF-36: Medical Outcomes Study 36-Item Short-Form Health Survey

### CBT competency and fidelity

The average ability score for the CFARS was 5.6 ± 0.4, and all general practitioners achieved scores ≥5. General practitioners scored higher for attention to CBT structure, treatment relationships, and providing feedback to patients. A total of 593 audio recordings were uploaded, and 143 evaluations (24%) were received. Each patient averaged 7.1 ± 0.5 points per class hour, which was in the range of very good to excellent.

### Changes in GAD-7 score

At baseline, the mean GAD-7 score of the intervention group was 7.57 ± 5.27 points and that of the control group was 7.62 ± 5.41 points, which did not significantly differ (t = 0.1, *p* = 0.92). After the 2-month intervention, the CBT group showed a significant reduction in GAD-7 score from baseline (t = − 4.245, *p* < 0.01), whereas the control group did not show a change (t = 0.059, *p* = 0.95; Table 2). The within-group effect size for the GAD-7 score change in the CBT group from baseline to 2 months was 1.27 (95% confidence interval [CI]: 0.88–1.71). GAD-7 scores at 2 months were significantly lower in the CBT group compared with those of the control group, with a large between-group effect size of 0.94 (95% CI: 0.83–1.25). The CBT group had a GAD-7 score that was 1.72 lower than the control group (1.47–1.99, *p* < 0.001). The subgroup analysis of the CBT group showed a significant reduction in GAD-7 score at 2 months in those with mild and moderate anxiety but not in those with severe anxiety (Table 3).

**Table 2:** GAD-7 and PHQ-9 scores before and after the intervention for each group

time		Intervention group (*n* = 230)	Control group (n = 224)	t	p
GAD-7	baseline	7.57 ± 5.27	7.62 ± 5.41	0.219	0.826
	2 months	5.93 ± 2.56	7.65 ± 5.38	6.092	<0.001
	t	−4.245	0.059		
	p	< 0.01	0.950		
PHQ-9	baseline	8.13 ± 6.01	8.00 ± 5.84	0.270	0.788
	2 months	6.02 ± 2.67	8.07 ± 5.91	7.345	<0.001
	t	−4.866	0.126		
	p	< 0.01	0.900		
SF-36	baseline	58.46 ± 12.71	59.11 ± 13.25	0.533	0.594
	2 months	74.31 ± 13.22	60.51 ± 13.76	10.898	< 0.001
	t	13.108	1.104		
	p	< 0.001	0.270		

GAD-7: Generalized Anxiety Disorder-7; PHQ-9: Patient Health Questionnaire-9; SF-36: Medical Outcomes Study 36-Item Short-Form Health Survey

**Table 3:** GAD-7 scores of patients with different degrees of anxiety and depression

group	Mild				Moderate				Severe			
	n	scores	t	p	n	scores	t	p	n	scores	t	p
Intervention group												
baseline	93	7.29 ± 1.21	8.056	< 0.01	52	11.99 ± 1.31	10.020	< 0.01	20	17.30 ± 1.58	0.839	0.41
2 months	93	5.54 ± 1.71			52	9.17 ± 1.55			20	16.89 ± 1.51		
Control group												
baseline	91	7.35 ± 1.29	0.084	0.93	53	11.90 ± 1.40	0.065	0.95	18	17.12 ± 1.62	0.640	0.53
2 months	91	7.37 ± 1.87			53	11.92 ± 1.74			18	16.77 ± 1.66		

GAD-7 consisted of seven items with a total score ranging from 0 to 21 points. Higher scores indicated greater severity of symptoms. A score of 0–4 points indicated no anxiety, 5–9 points indicated mild anxiety, 10–14 points indicated moderate anxiety, and 15–21 points indicated severe anxiety

### Changes in PHQ-9 score

The mean PHQ-9 scores of the intervention and control groups were 8.13 ± 6.01 points and 8.00 ± 5.84 points, respectively. The scores did not differ significantly between the two groups (t = 0.295, *p* = 0.77). After the 2-month intervention, PHQ-9 scores of the CBT group were lower than those at baseline (t = − 4.866, *p* < 0.01), whereas in the control group, there was no change in PHQ-9 score (t = 0.126, *p* = 0.90; Table 2). The within-group effect size for the change in PHQ-9 score from baseline to 2 months for the CBT group was 1.31 (95% CI: 0.93–1.85). PHQ-9 scores at 2 months were significantly lower in the CBT group compared with those of the control group, with a large between-group effect size of 0.96 (95% CI: 0.82–1.32). The CBT group had a PHQ-9 score that was 2.05 lower than that of the control group (1.74–2.37, *p* < 0.001). Results showed that CBT reduced depression symptoms in patients. The subgroup analysis of the PHQ-9 score indicated that CBT reduced scores in patients with mild and moderate depression at 2 months but not in those with severe or very severe depression (Table 4).

**Table 4:** PHQ-9 scores of patients with different degrees of anxiety and depression

group	Mild				Moderate				Moderate-Severe				Severe			
	n	scores	t	p	n	scores	t	p	n	scores	t	p	n	scores	t	p
Intervention group																
baseline	100	7.01 ± 1.47	13.588	< 0.01	43	11.88 ± 1.12	8.852	< 0.01	20	17.65 ± 1.11	1.510	0.14	8	23.10 ± 1.44	0.796	0.44
2 months	100	4.42 ± 1.22			43	8.39 ± 2.33			20	16.86 ± 2.06			8	22.32 ± 2.37		
Control group																
baseline	103	6.84 ± 1.39	0.769	0.44	39	12.13 ± 1.47	1.727	0.09	19	17.48 ± 1.26	0.307	0.76	7	22.89 ± 1.52	0.194	0.85
2 months	103	6.99 ± 1.41			39	11.58 ± 1.34			19	17.75 ± 1.35			7	23.06 ± 1.75		

PHQ-9 consisted of nine items with a total score ranging from 0 to 27 points. Higher scores indicated greater severity of symptoms. A score of 0–4 points indicated no depression, 5–9 points indicated mild depression, 10–14 points indicated moderate depression, 15–19 points indicated heavy depression, and 20–27 points indicated severe depression

### Changes in quality of life

Changes in SF-36 score in the CBT and control groups are shown in Table 2. At baseline, there was no significant difference in the mean SF-36 score between the two groups (t = 0.533, *p* = 0.594). After the 2-month intervention, the mean SF-36 scores of the CBT group were lower than those at baseline (t = 13.108, *p <* 0.01), whereas the control group showed no change (t = 1.104, *p* = 0.27). The CBT group had a total SF-36 score that was 10.7 lower than that of the control group (95% CI: 7.9–13.5, *p* < 0.001). The within-group effect size for the change in SF-36 score from baseline to 2 months for the CBT group was 1.30 (95% CI: 0.86–1.84). SF-36 scores at 2 months were significantly lower in the CBT group compared with those of the control group (t = 38.943, *p* < 0.001), with a between-group effect size of 0.85 (95% CI: 0.71–1.05).

### Adverse events

Eleven people in the CBT group and 27 people in the control group were re-hospitalized within 2 months because of pulmonary tuberculosis. The difference between the two groups was statistically significant (χ^2^ = 7.82, *p* < 0.01).

## Discussion

To the best of our knowledge, this is the first study to demonstrate that general practitioners can be trained to effectively deliver CBT for individuals with pulmonary tuberculosis. Results showed that CBT significantly reduced GAD-7 and PHQ-9 scores and increased SF-36 scores in patients at 2 months, which was not observed in those who received usual care. Our study showed that CBT can alleviate anxiety and depression symptoms and improve the quality of life of pulmonary tuberculosis patients.

CBT is an evidence-based intervention for the treatment of mental illness that includes psychological education, coping skills, cognitive reconstruction, catharsis, and homework exercises [32]. The intervention reshapes an individual’s long-term distorted thoughts, beliefs, and behaviors. Thus, the therapy has been recommended as the preferred non-drug therapy in the treatment of mental illness [33] and has been implemented widely for the treatment of mental illnesses combined with physical diseases [12, 14, 34]. On this basis, CBT is expected to be effective for the treatment of anxiety and depression as the two most common psychological problems in patients with pulmonary tuberculosis [4, 5]; this was confirmed by our study. Although we used the same anxiety and depression assessment scales as previous studies, the level of improvement differed. Sun H [34] find improvement in anxiety was observed in patients treated with CBT relative to controls without CBT, but depression and quality of life improvement were not observed. One reason may be related to differences in comorbid basic diseases. An alternative reason may be related to the intensity of the intervention. Because the DOTS strategy used for patients with pulmonary tuberculosis requires general practitioners to interact with patients daily to ensure medication compliance, we required general practitioners to complete CBT within 1 month and provide further therapy to strengthen in the second month. While not excluding general practitioners face patients to take medication every day, patients ask questions and general practitioners give answers to the patients. Therefore, the intensity of our intervention was greater than that of other studies.

We found different degrees of anxiety and depression across patients with pulmonary tuberculosis, and improvements in anxiety and depression varied following CBT. Only pulmonary tuberculosis patients with mild and moderate anxiety and depression showed reduced symptoms following CBT; CBT had no effect on patients with severe levels of anxiety and depression. About one-third of depressed patients are reported to be ineffective to conventional antidepressant treatment, psychotherapy and cognitive-behavioral therapy, called refractory depression [35]. Results indicated that patients with severe anxiety and depression require specialist intervention or supplementary medication. Therefore, we recommend that pulmonary tuberculosis patients who also experience severe anxiety or depression be promptly referred to a psychiatric department of a specialist or general hospital for treatment.

Quality of life is a comprehensive reflection of a person’s health, psychological status, independence level, social relations, personal beliefs, and other indicators. Anxiety and depression are the main risk factors for the poor quality of life in patients with pulmonary tuberculosis [6]. Therefore, psychological interventions that can reduce symptoms of anxiety and depression are likely to improve the quality of life of patients [36]. This is consistent with the results of our study. Because of the infectiousness of pulmonary tuberculosis, patients often feel ashamed or are unwilling to interact with society [37]. CBT can reduce the negative emotions of patients, which is equivalent to providing patients with social support. Some evidence indicates social support is also associated with depressive and anxiety symptoms and improvements in effect [38]. Relaxation training can improve certain aspects of a patient’s personality, enhance positive qualities, and adjust poor habits and attitudes [39], which in turn, will reduce anxiety and depression symptoms and improve quality of life [40].

This study has several shortcomings. A subjective scale was used for the assessment of anxiety and depression, which may have an impact on the results. Furthermore, this study was not a second-level community randomized controlled trial, because there were fewer patients in the same time period from each community and individual intervention was used, rather than group intervention. To prevent contamination.

"we chose the same management approach for all patients across all communities. In theory, the effect of the individual intervention should be superior to the group intervention. However, because of the limited observation time, we did not compare the effects of pulmonary tuberculosis patients between intervention groups and control groups. Nevertheless, the negative event results suggested that the effects of the intervention group were better than that of the control group.

This study showed that CBT is effective in reducing symptoms of depression and anxiety in pulmonary tuberculosis patients. CBT can encourage patients to face the disease, return to society, and improve their quality of life. Because of the high prevalence of anxiety and depression in pulmonary tuberculosis patients, alongside the ease of use of the GAD-7 and PHQ-9, we recommend general practitioners to screen patients with pulmonary tuberculosis for anxiety and depression and promote the use of CBT as an intervention.

## Data Availability

The datasets used and/or analysed during the current study were available from the corresponding author on reasonable request.

## References

[1] World Health Report 2001: Global Tuberculosis Control. http://apps.who.int/iris/bitstream/10665/63835/4/WHO_CDS_TB_2001.287.pdf.

[2] World Health Organization (2019). Global tuberculosis report, 2019. WHO/CDS/TB/2019.15.

[3] Ambaw F, Mayston R, Hanlon C, Alem A (2017). Burden and presentation of depression among newly diagnosed individuals with TB in primary care settings in Ethiopia. BMC Psychiatry.

[4] Duko B, Bedaso A, Ayano G (2020). The prevalence of depression among patients with tuberculosis: a systematic review and meta-analysis. Ann General Psychiatry.

[5] Wang XB, Li XL, Zhang Q (2018). A survey of anxiety and depressive symptoms in pulmonary tuberculosis patients with and without tracheobronchial tuberculosis. Front Psychiatry.

[6] Dos Santos AP, Lazzari TK, Silva DR (2017). Health-related quality of life, depression and anxiety in hospitalized patients with tuberculosis. Tuberc Respir Dis.

[7] Ambaw F, Mayston R, Hanlon C, Medhin G, Alem A (2018). Untreated depression and tuberculosis treatment outcomes, quality of life and disability, Ethiopia. Bull World Health Organ.

[8] Ruiz-Grosso P, Cachay R, de la Flor A, Schwalb A, Ugarte-Gil C (2020). Association between tuberculosis and depression on negative outcomes of tuberculosis treatment: a systematic review and meta-analysis. PLoS One.

[9] Tola HH, Shojaeizadeh D, Tol A (2016). Psychological and educational intervention to improve tuberculosis treatment adherence in Ethiopia based on health belief model: a cluster randomized control trial. PLoS One.

[10] Peddireddy V (2016). Quality of life, psychological interventions and treatment outcome in tuberculosis patients: the Indian scenario. Front Psychol.

[11] Husain MO, Dearman SP, Chaudhry IB, Rizvi N, Waheed W (2008). The relationship between anxiety, depression and illness perception in tberculosis patients in Pakistan. Clin Pract Epidemiol Ment Health.

[12] Cully JA, Stanley MA, Petersen NJ (2017). Delivery of brief cognitive behavioral therapy for medically ill patients in primary care: a pragmatic randomized clinical trial. J Gen Intern Med.

[13] Pateraki E, Morris PG (2018). Effectiveness of cognitive behavioural therapy in reducing anxiety in adults and children with asthma: a systematic review. J Asthma.

[14] Xu C, Dong Z, Zhang P (2021). Effect of group cognitive behavioural therapy on psychological stress and blood glucose in people with type 2 diabetes mellitus: a community-based cluster randomized controlled trial in China. Diabet Med.

[15] Beck AT (1970). Cognitive therapy: nature and relation to behavior therapy. Behav Ther.

[16] Ellis A (1962). Reason and emotion in psychotherapy.

[17] World Health Organization (1997). Treatment of tuberculosis. Guidelines for national programmes. WHO report.

[18] Moher D, Schulz KF, Altman DG (2001). The CONSORT statement: revised recommendations for improving the quality of reports of parallel-group randomised trials. Lancet..

[19] Chinese Society for Tuberculosis, Chinese Medical Association (CSTB) (2013). Guidelines on the diagnosis and treatment of pulmonary tuberculosis. Chin Prac J Rural Doc.

[20] Donner A, Klar N (2000). Design and analysis of cluster randomization trials in health research.

[21] Westbrook D, Kennerley H, Kirk J (2011). An introduction to cognitive behaviour therapy: skills and applications.

[22] Gold JA, Spurlin D (2001). Ask, assess, advise, assist, arrange are keys to smoking cessation. WMJ..

[23] Mannix KA, Blackburn IM, Garland A, Gracie J, Moorey S, Reid B, Standart S, Scott J (2006). Effectiveness of brief training in cognitive behaviour therapy techniques for palliative care practitioners. Palliat Med.

[24] Spitzer RL, Kroenke K, Williams JB, Löwe B (2006). A brief measure for assessing generalized anxiety disorder: the GAD-7. Arch Intern Med.

[25] Gong Y, Zhou H, Zhang Y, Zhu X, Wang X, Shen B, Xian J, Ding Y (2021). Validation of the 7-item generalized anxiety disorder scale (GAD-7) as a screening tool for anxiety among pregnant Chinese women. J Affect Disord.

[26] Kroenke K, Spitzer RL, Williams JB (2001). The PHQ-9:validity of a brief depression severity measure. J Gen Intern Med.

[27] Liu ZW, Yu Y, Hu M, Liu HM, Zhou L, Xiao SY (2016). PHQ-9 and PHQ-2 for screening depression in Chinese rural elderly. PLoS One.

[28] Wang Y, Lii J, Lu F (1998). Measuring and assessing the quality of life of patients with pulmonary tuberculosis. Zhonghua Jie he he Hu xi Za Zhi.

[29] Zhu Z, Zhu D, Jiang Y, Lin Y, Yang Y, Luan W (2021). Cross-sectional study on the SF-36, the general self-efficacy, the social support, and the health promoting lifestyle of the young elderly in a community in Shanghai, China. Ann Palliat Med.

[30] Cully JA, Mignogna J, Stanley MA (2012). Development and pilot testing of a standardized training program for a patient-mentoring intervention to increase adherence to outpatient HIV care. AIDS Patient Care STDs.

[31] Cohen J (1988). Statistical power analysis for the behavioral sciences.

[32] Shahnavaz S, Hedman-Lagerlöf E, Hasselblad T, Reuterskiöld L, Kaldo V, Dahllöf G (2018). Internet-based cognitive behavioral therapy for children and adolescents with dental anxiety: open trial. J Med Internet Res.

[33] Uchendu C, Blake H (2017). Effectiveness of cognitive-behavioural therapy on glycaemic control and psychological outcomes in adults with diabetes mellitus: a systematic review and meta-analysis of randomized controlled trials. Diabet Med.

[34] Sun H, Huang H, Ji S (2019). The efficacy of cognitive behavioral therapy to treat depression and anxiety and improve quality of life among early-stage breast Cancer patients. Integr Cancer Ther.

[35] Fava M (2003). Diagnosis and definition of treatment-resistant depression. Biol Psychiatry.

[36] Dar SA, Shah NN, Wani ZA, Nazir DA (2019). a prospective study on quality of life in patients with pulmonary tuberculosis at a tertiary care hospital in Kashmir, northern India. Indian J Tuberc.

[37] Molla A, Mekuriaw B, Kerebih H (2019). Depression and associated factors among patients with tuberculosis in Ethiopia: a cross-sectional study. Neuropsychiatr Dis Treat.

[38] Lu Q, Chen L, Shin LJ, Wang C, Dawkins-Moultin L, Chu Q, Loh A, Young L, Wang C (2021). Improvement in quality of life and psychological well-being associated with a culturally based psychosocial intervention for Chinese American breast cancer survivors. Support Care Cancer.

[39] Kumar S, Nayak RR, Devi SK (2015). Effectiveness Jacobson’s progressive muscle relaxation technique (PMRT) to relieve anxiety among alcoholic patients MHI, SCB, Cuttack, Odisha. J Nurs Health Sci.

[40] Zhao L, Wu H, Zhou X, Wang Q, Zhu W, Chen J (2012). Effects of progressive muscular relaxation training on anxiety, depression and quality of life of endometriosis patients under gonadotrophin-releasing hormone agonist therapy. Eur J Obstet Gynecol Reprod Biol.

